# Incidental finding of isolated uterine cervix tuberculosis with successful management: A case report

**DOI:** 10.1016/j.ijscr.2024.110693

**Published:** 2024-11-29

**Authors:** Teketel Tadesse Geremew, Woldie Jember Zewdie, Noh Abidirkadir Seid, Tigist Gutema

**Affiliations:** a Department of Pathology, Hawassa University Comprehensive Specialized Hospital, Hawassa, Sidama, Ethiopia; b Department of Pathology, Worabe Comprehensive Specialized Hospital, Ethiopia; c Department of Internal medicine, Hawassa University Comprehensive Specialized Hospital, Hawassa, Sidama, Ethiopia

**Keywords:** Histopathology, Tuberculosis, Cervical cancer, Cervix

## Abstract

**Introduction and importance:**

Cervical tuberculosis is an uncommon condition which accounts for 0.1–0.65 % of all cases of tuberculosis (TB). In women with genital TB, four major presenting complaints are described with varying frequencies: infertility, abnormal bleeding, pelvic pain, and amenorrhea. As with other parts of the female genital tract; there are no macroscopic changes in the cervix that are specific for TB.

The diagnosis of TB is based on the identification of *M. tuberculosis*. The treatment of tuberculosis of the cervix is essentially based on antibacillary bactericidal drugs and surgical management based on indication. This case is important because there are few case reports in the world, and it is among the few case to be reported from Ethiopia.

**Case presentation:**

Here we present a 45-year-old female patient presented with odorless whitish vaginal discharge and pelvic pain, she was diagnosed with cervical cancer clinically but histomorphologic examination confirms the diagnosis of primary uterine cervix tuberculosis.

**Clinical discussion:**

TB is a bacterial infection frequently seen in less developed countries. It is a frequent cause of chronic pelvic inflammation and infertility. Tuberculosis of the cervix may occur as a primary infection or secondary (in which case the primary focus would have healed). Approximately one third of cases are culture negative. Therefore, the presence of typical granulomata is sufficient for diagnosis if other causes of granulomatous cervicitis are excluded or a primary focus identified.

**Conclusion:**

Uterine cervix is an uncommon genital location for tuberculosis. There are no macroscopic changes in the cervix that are specific for TB and it can mimic cervical cancer clinically. A definitive diagnosis of TB requires isolation of tubercle bacilli. It should be considered as a differential diagnosis in patients presenting with non-specific lower genital tract complaints. Close collaborations between clinicians and diagnosticians is essential in accurately diagnosing and managing this rare condition.

## Highlights

• Cervical tuberculosis is an uncommon condition which accounts for 0.1-0.65% of all cases of tuberculosis (TB).
• As with other parts of the female genital tract; there are no macroscopic changes in the cervix that are specific for TB.
• The diagnosis of TB is based on the identification of *M. tuberculosis*.
• The treatment of tuberculosis of the cervix is essentially based on antibacillary bactericidal drugs.

## 1 Introduction

Cervical tuberculosis accounts for 0.1–0.65 % of all cases of tuberculosis (TB) [1,2]. Tuberculosis more frequently affects the upper genital tract-namely, the fallopian tubes and endometrium [3]. TB of the cervix is present in about 5 % of the cases [4]. In women with genital TB, four major presenting complaints are described with varying frequencies: infertility, abnormal bleeding, pelvic pain, and amenorrhea [5]. As with other parts of the female genital tract; there are no macroscopic changes in the cervix that are specific for TB. The cervix may appear normal or inflamed, and its condition may resemble invasive carcinoma, both grossly and with the colposcope. The most common type is the ulcerative form, although papillomatous and miliary forms may also occur [5]. The diagnosis of TB is based on the identification of *M. tuberculosis* [6]. The treatment of tuberculosis of the cervix is essentially based on antibacillary bactericidal drugs (Rifampicin, Isoniazid, pyrazinamide, streptomycin) and antibacillary bacteriostatic drugs (Ethambutol, Ethionamide) [7]. We present such a case due to the rarity of this condition and that it clinically mimics carcinoma of cervix.

This case report is written following the SCARE criteria [8].

## 2 Case presentation

Our case is a 45-year-old female patient from the Ethiopia who has presented with vaginal odorless smelling discharge of 1 year duration; initially it was intermittent and minimal in amount; later it became continuous and significant in amount. She has pelvic pain and loss of appetite. She is Para v mother with no history of miscarriage or abortion. Otherwise, the patient didn't have any history of trauma to the site. She doesn't have any history of diabetes mellitus, hypertension, or immunocompromization. She doesn't have history of cough or TB treatment. She doesn't have any contact with known TB or TB treated patients. She is vaccinated for HPV virus as protocol.

### 2.1 Physical findings

Vital signs were all within the normal limits.

#### 2.1.1 Respiratory examination

Unremarkable with good air entry bilaterally.

#### 2.1.2 Genitourinary examination

Digital pelvic examination and bimanual examination revels odorless whitish discharge. Otherwise, no mass identified.

With clinical suspicions of early cervical cancer and to rule out precursor cervical lesions, investigations were done on investigation.

Full blood count (WBC-9.8 × 10 9/L, Hgb-12.4 g/dl, platelet- 305 × 10 9/L), ESR-65 mm/h, liver and renal function tests were within the normal range. Antibody tests for HIV and VDRL infections were negative and the chest x-ray was normal.

#### 2.1.3 Abdominopelvic ultrasound

Unremarkable with normal fallopian tube and no mass or lesion identified. A pap smear was done, and the finding was atypical glandular cells (favor benign) and punch biopsy were advised.

**Fig. 1:**
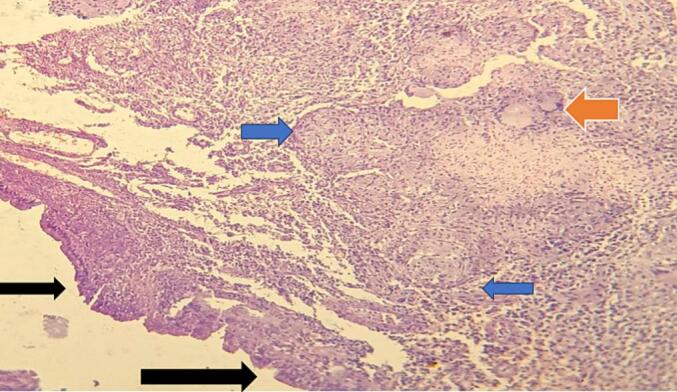
Low power view(4×) shows cervical tissue containing bland endocervical (blue arrow) and ectocervical epithelium (black arrow) composed of subepithelial granuloma (blue arrow) and langhan's giant cell (orange arrow). (For interpretation of the references to colour in this figure legend, the reader is referred to the web version of this article.)

**Fig. 2:**
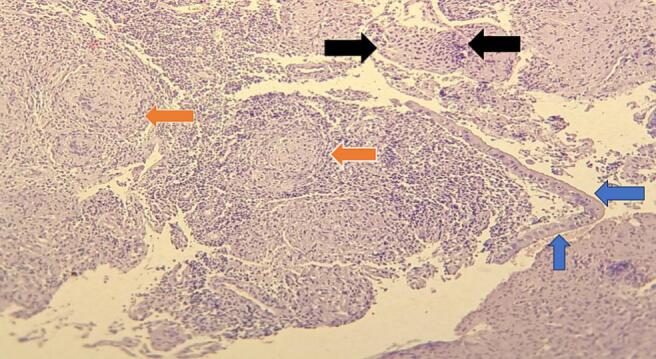
Low power view(4×) shows cervical tissue lined by bland endocervical epithelium (black arrow) composed of subepithelial granuloma (orange arrow).

**Fig. 3:**
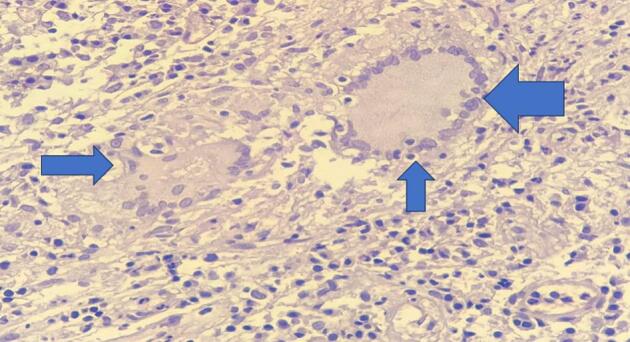
High power view(40×) shows horse shoe like langhan's giant cells (blue arrow). (For interpretation of the references to colour in this figure legend, the reader is referred to the web version of this article.)

**Fig. 4:**
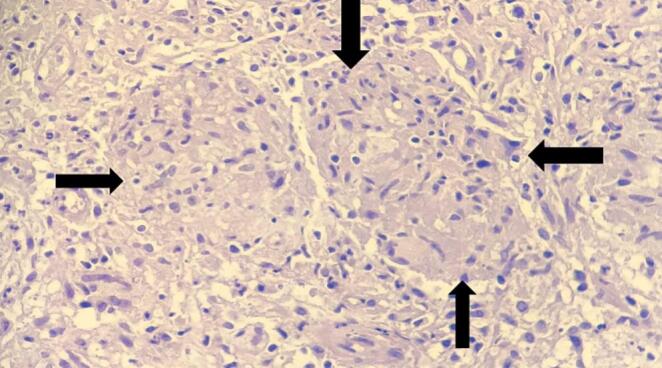
High power view(40×) shows nodular aggregates of activated epithelioid cells forming a granuloma (black arrow).

A gross evaluation of the biopsy specimen revealed multiple gray-white to gray-brown cervical tissue fragments measuring 2 cm in aggregate, all are embedded. The lesion's microscopic examination with routine H&E stain revealed a tissue with bland stratified squamous and endocervical columnar epithelium with subepithelial repetitive granuloma formation, langhan's type of giant cells and dusty granular necrosis seen. The conclusion of the biopsy was uterine cervix tuberculosis (Fig. 1, Fig. 2, Fig. 3, Fig. 4). Scanned acid-fast bacilli were found on microscopy. Special staining for fungus was carried out which was negative.

She was put on antitubercular treatment for six months, and then biopsy was taken from cervix which revealed no evidence of tuberculosis. The vaginal discharge subsided, and lower abdominal pain improved.

## 3 Discussion

Tuberculosis is an ancient disease that has been a major cause of suffering and death for many years. TB has been the leading cause of death among infectious diseases. Successful antibiotic treatment only became possible in 1948. In humans, tuberculosis is caused by the bacillus *Mycobacterium tuberculosis*. It mainly affects the lungs, although it can affect any organ in the body (extrapulmonary TB) [3]. The tubercle bacilli were first described by Robert Koch in 1882.TB is a bacterial infection frequently seen in less developed countries. Tuberculosis commonly (77 %) affects the respiratory (pulmonary) system causing cough as the most common symptom but genital organ involvement is rare. TB involvement in the female genital tract in almost all cases is secondary to extragenital tuberculosis. The fallopian tubes are affected most commonly (90 %), followed by the endometrium (50 %) and the ovaries (10–30 %). The cervix is rarely involved and accounts for 5 of the cases of genital tract [9]. Tuberculosis of the cervix may occur as a primary infection or secondary (in which case the primary focus would have healed) [10,11]. Our patient has primary infection with cervical tuberculosis. As a general rule, genital tract tuberculosis involvement may be through: blood (90 %), spread from other organs (e.g. tuberculous peritonitis), lymph node involvement, and “vertical” spread through intercourse with an infected partner [11].

The common presentations in TB of the female genital tract are amenorrhea, menstrual irregularities, infertility, vaginal discharge and postmenopausal bleeding. It is a frequent cause of chronic pelvic inflammation and infertility. Our patient presented with vaginal discharge and pelvic pain. The gross appearance of the tuberculous cervix is highly variable. It may present as papillary, ulcerative, interstitial, miliary, endocervical or polypoid forms [11,12].

Isolation of the mycobacterium is the gold standard for diagnosis. Approximately one third of cases are culture negative. Therefore, the presence of typical granulomata is sufficient for diagnosis if other causes of granulomatous cervicitis are excluded or a primary focus identified [13]. Our patient has punch biopsy consistent with tuberculosis and scanned acid-fast bacilli were found on microscopy. Other causes of granulomatous lesions need to be ruled out such as lymphogranuloma venereum, sarcoidosis, schistosomiasis and foreign body cell granulomas secondary to a non-absorbable suture or cotton [14].

The treatment of tuberculosis of the cervix is essentially based on antibacillary bactericidal drugs (Rifampicin, Isoniazid, pyrazinamide, streptomycin) and antibacillary bacteriostatic drugs (Ethambutol, Ethionamide). The efficacy and safety of treatment should be carefully monitored. The surgical management of uterine adhesions currently imposes a hysteroscopy to improve fertility. The use of surgical treatment should be reserved for the management of complications (fistulas or abscesses) or in case of resistance or relapse in well conducted medical treatment. This treatment must be preceded and followed by drug treatment. The post treatment surveillance of tuberculosis of the cervix requires regular speculum examination and control biopsies, if necessary [7].

## 4 Conclusions

Uterine cervix is an uncommon genital location for tuberculosis. There are no macroscopic changes in the cervix that are specific for TB and it can mimic cervical cancer clinically. A definitive diagnosis of TB requires isolation of tubercle bacilli. It should be considered as a differential diagnosis in patients presenting with non-specific lower genital tract complaints. Close collaborations between clinicians and diagnosticians is essential in accurately diagnosing and managing this rare condition.

## Abbreviations

TB: Tuberculosis
ESR: Erythrocyte Sedimentation Rate
HIV: Human Immunodeficiency Virus
VDRL: Venereal Disease Research Laboratory test

## Author contribution

Teketel Tadesse Geremew, MD - Study concept and design, writing and drafting the paper, literature review and editing and critical review of the paper.

Woldie Jember Zewdie and Noh Abidirkadir Seid, MD -Involved in acquisition of data, literature review of the paper, writing the paper, editing and critical review of the paper.

Tigist Gutema Tesgera, MD: literature review of the paper, writing the paper, editing and critical review of the paper.

## Consent

Written informed consent was obtained from the patient, by their native language, for publication of non-identifying information including accompanying intraoperative images. A copy of the written consent is available for review by the Editor-in-Chief of this journal on request.

## Ethics approval

No ethics approval was needed as the case was encountered incidentally and it doesn't involve any human or animal.

## Guarantor

Teketel Tadesse Geremew, MD.

## Research registration number

None.

## Funding

No funding was provided for this case report.

## Declaration of competing interest

The authors declare that there are no conflicts of interest on this case report.
